# Ontogenetic transition from specialized root hairs to specific root-fungus symbiosis in the dominant Mediterranean seagrass *Posidonia oceanica*

**DOI:** 10.1038/s41598-018-28989-4

**Published:** 2018-07-17

**Authors:** Ondřej Borovec, Martin Vohník

**Affiliations:** 1 Department of Mycorrhizal Symbioses, Institute of Botany, Czech Academy of Sciences, Průhonice, CZ 25243 Czech Republic; 2 Department of Experimental Plant Biology, Faculty of Science, Charles University, Prague, CZ 12843 Czech Republic

## Abstract

Terrestrial plants typically take up nutrients through roots or mycorrhizae while freshwater plants additionally utilize leaves. Their nutrient uptake may be enhanced by root hairs whose occurrence is often negatively correlated with mycorrhizal colonization. Seagrasses utilize both leaves and roots and often form root hairs, but seem to be devoid of mycorrhizae. The Mediterranean seagrass *Posidonia oceanica* is an exception: its adults commonly lack root hairs and regularly form a specific association with a single pleosporalean fungus. Here we show that at two sites in the southern Adriatic, all its seedlings possessed abundant root hairs with peculiar morphology (swollen terminal parts) and anatomy (spirally formed cell walls) as apparent adaptations for better attachment to the substrate and increase of breaking strain. Later on, their roots became colonized by dark septate mycelium while root hairs were reduced. In adults, most of terminal fine roots possessed the specific fungal association while root hairs were absent. These observations indicate for the first time that processes regulating transition from root hairs to root fungal colonization exist also in some seagrasses. This ontogenetic shift in root traits may suggests an involvement of the specific root symbiosis in the nutrient uptake by the dominant Mediterranean seagrass.

## Introduction

Mineral nutrition of vascular terrestrial plants typically depends on roots that transport dissolved elements from the rhizosphere, a relatively small volume of the soil surrounding the root (i.e., the zone of ca. 1–2 mm around the root). In order to facilitate nutrient uptake from the rhizosphere many plants form root hairs, unicellular tubular rhizodermal structures that vastly increase root surface area, root absorption capacity and influx of nutrients^1,2^. Nutrient uptake through root hairs may be further increased by secretion of root exudates^3^ which also play an important role in mediating interactions between roots and symbiotic soil organisms such as the mutualistic mycorrhizal fungi or nitrogen-fixing bacteria^4^. In addition to their role in plant nutrition, roots play an important role in anchoring plants in the substrate, storage of nutrients, clonal growth/vegetative propagation, aeration of the rhizosphere, etc.^5^.

In most terrestrial ecosystems, nutrients are bound in recalcitrant substrates not readily available to plant roots. Under these circumstances, the great majority of vascular plants form dual root-fungus organs called mycorrhizae, because mycorrhizal fungi can access these substrates and provide the released nutrients to their host plants in exchange for photosynthetic carbon^6^. The extraradical mycelium of mycorrhizal fungi extends far beyond the rhizosphere and forms the so-called mycorrhizosphere – a volume of soil under a combined influence of the root and the emerging fungal hyphae^7^. Interestingly, many obligately mycotrophic plants (i.e., dependent in mineral nutrient uptake on mycorrhizal fungi) down-regulate production of root hairs upon establishment of the mycorrhizal symbiosis and many notoriously non-mycorrhizal plant guilds such as carnivorous plants and plants forming cluster or dauciform roots possess very dense root hair cover^8^.

Submerged freshwater macrophytes have adapted a rather different strategy – although nutrient uptake through roots may still play an important role in their nutrition, especially in water with low nutrient content^9^, most of them acquire a significant part of mineral nutrients from the water column through their leaves^10^. Similarly to terrestrial plants, freshwater macrophytes may increase nutrient uptake through the roots hairs^11,12^, may engage in symbioses with arbuscular mycorrhizal fungi^13,14^ and there seems to be a negative correlation between mycorrhizal colonization and presence of root hairs^15,16^.

Also seagrasses, the only vascular plants adapted to permanent life in the marine ecosystem, are capable of acquiring significant amounts of nutrients through their leaves^17,18^, but the influx through roots may still be essential especially in the species inhabiting oligotrophic areas^19,20^. Despite that most seagrasses produce root hairs^12,21^ their functioning has been only rarely investigated and is not fully understood. The growth of root hairs may be triggered by low nitrogen concentrations in the substrate suggesting engagement in the nutrient uptake^12^. Additionally, root hairs are able to attach to the substrate particles thus helping to significantly enhance the anchoring strength especially in the early phases of the seagrass development^22,23^.

Unlike most terrestrial plants, seagrasses seem to be devoid of mycorrhizae^24^ but similarly to terrestrial plants, their roots may be colonized by endophytic fungi. Fungal endophytes grow within living plant tissues often forming an unapparent infection lacking specific interfaces for nutrient transfer with the host^25^ and especially do not form mycorrhizae or cause any obvious disease symptoms^26^. Many of the so far reported seagrass fungal endophytes represent facultative marine fungi which also readily occur in terrestrial ecosystems, but some are strictly marine fungal genera^27^ which can partly act as endophytes but mostly grow as saprobes and decompose seagrass tissues.

Next to nothing is so far known about the ecology, functioning and significance of seagrass fungal symbionts (=mycobionts). However, the recent discovery of a specific association between the roots of the seagrass *Posidonia oceanica* and a yet undescribed mycobiont from Pleosporales (Ascomycota) suggests that ecologically significant relationships may exist also between seagrasses and their root-associated fungi^28–30^. From the anatomical/morphological perspective, this association can be assigned as a unique morphotype of the so-called dark septate endophytic (DSE) symbiosis^30^. DSE fungi are ubiquitous in the roots of terrestrial plants^31,32^ where they form more or less uniform hyphal structures, especially the characteristic intracellular microsclerotia^25,33^ and perform various functions along the mutualism-parasitism continuum^34^. However, their role in the marine environment has not yet been investigated.

*Posidonia oceanica* (L.) Delile is endemic to the Mediterranean Sea where it inhabits shallow depths from ca 0.5 to ca. 40 meters, forming typical dense meadows which may cover large areas (up to tens of square kilometers) and living hundreds to thousands of years^35^. The Mediterranean Sea is generally oligotrophic with phosphorus being the most important limiting nutrient, although closely followed by nitrogen^36^. Under these conditions, leaf nutrient uptake alone is insufficient for successful growth and maturation of *P. oceanica* and the seagrass has to rely also on the influx of nutrients through its root system^20^. *Posidonia oceanica* typically colonizes coarse sandy to rocky bottoms, i.e., mostly mineral substrates, but with progressing time, it forms the so-called matte, a compact substance formed by degrading leaves, rhizomes and roots mixed with sediments and the seabed substrate, which can be up to several meters thick. The matte typically stores large amounts of organically-bound nutrients and provides significant primary source of carbon and mineral elements to the whole meadow ecosystem^37^. However, the latter is typically non-available to plant roots without the aid of symbiotic organisms or even non-symbiotic saprobes which decompose organic substrates and release mineral nutrients into the (myco-)rhizosphere^38^.

In contrast to most seagrasses, *P. oceanica* has been traditionally quoted as a typical species which lacks root hairs^24,39^ but some more recent studies indicate that these important root structures can be occasionally formed, although usually at low densities, also in this seagrass^30,40^. Most recently, Badalamenti *et al*.^23^ reported that *P. oceanica* seedlings were capable of forming dense root hair covers as an adhesion adaptation to rocky substrates. However, to our knowledge, nothing is so far known about the possible involvement of root hairs in the nutrient uptake in *P. oceanica* and this holds true also for its root mycobionts^27,29,30^.

With respect to reproduction, *P*. *oceanica* mostly relies on vegetative propagation^35,41^ as its flowering is thought to be rare and irregular^42,43^. However, some studies indicate that *P. oceanica* flowering events may be more frequent than usually thought^44–46^ and their frequency is likely to increase due to warming of the Mediterranean^43^. *Posidonia oceanica* generative reproductive success is usually low (but see^46^), being significantly reduced already at the pre-dispersal phase by factors like seed abortion and herbivory^47^. The seed dispersal and seedling establishment (especially during the first year) phases represent additional bottlenecks eventually determining the outcome of sexual reproduction of this seagrass^46,48–50^.

During sampling of *P. oceanica* roots for screening of their fungal colonization, two populations of the seagrass seedlings were discovered in the southern Adriatic Sea off Montenegro; intriguingly, their roots often seemed to be covered with root hairs. Here we focused on their selected root traits (root hair presence/density and morphology, fungal colonization) and compared these traits with those of a neighboring adult plant population. Additionally, we screened the effect of substrate type (sandy vs. silty) on the same traits in adult plants from two other localities in the same region. Based on our previous observations from the NW Mediterranean^28–30^ as well as *in situ* observations at the localities in Montenegro, we hypothesized that the roots of young seedlings would have dense root hair cover but would lack the typical fungal colonization, and that this scenario would be reversed in the adult plants, i.e., they would lack root hairs but would be densely colonized by dark septate mycelium. Additionally, we hypothesized that the substrate type would significantly affect both root hair density and root fungal colonization.

## Results

### Seedlings

**Figure 1:**
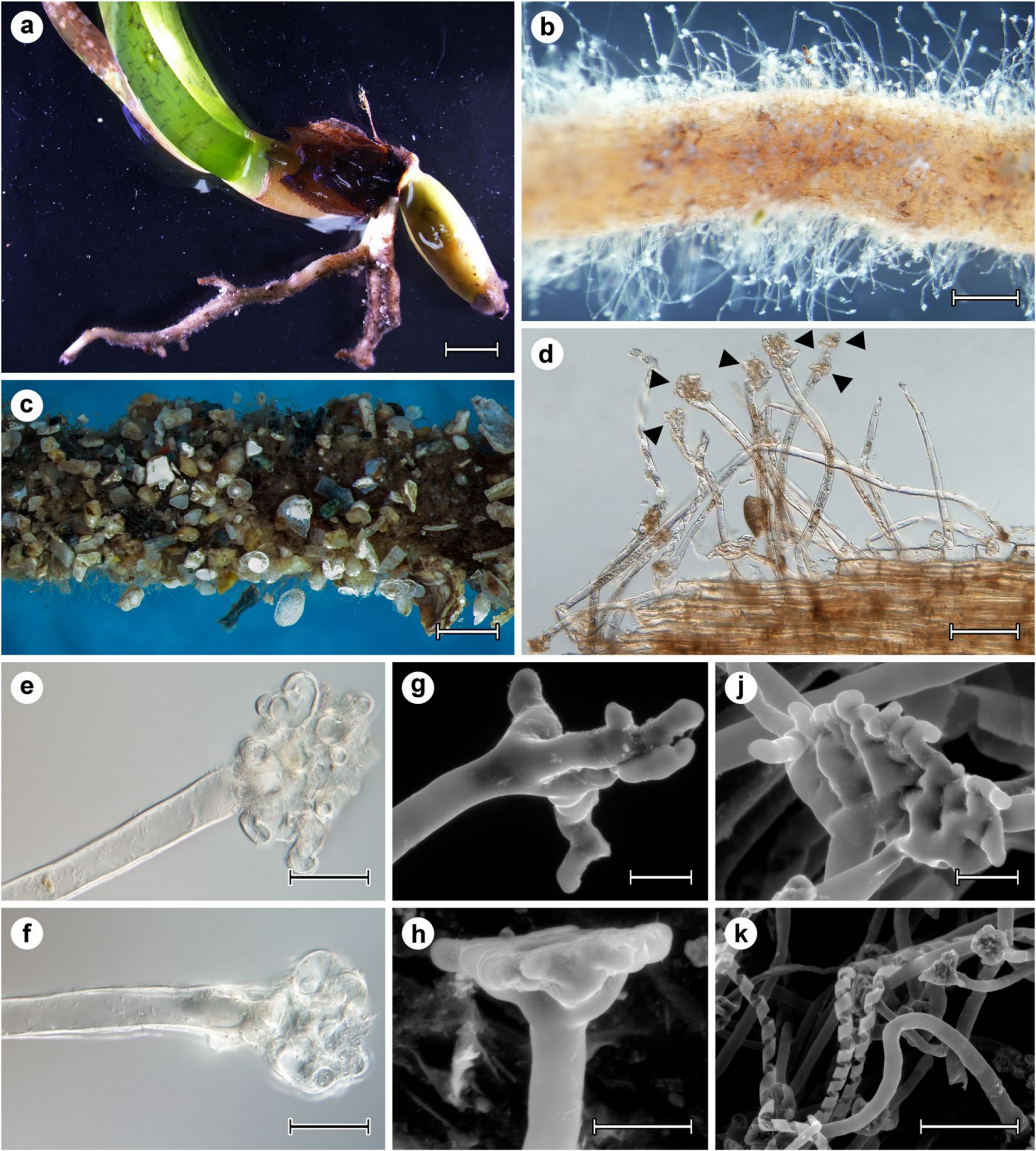
Root hairs in *Posidonia oceanica* seedlings. (**a**) The lower part of a *P. oceanica* seedling with relatively thick roots densely covered with root hairs. (**b**) Detail of a *P. oceanica* seedling root with numerous root hairs. Stereomicroscope, bar = 0.5 mm. (**c**) Substrate particles attached to the numerous root hairs. Stereomicroscope, bar = 1 mm. (**d**) Terminal parts of the root hairs possess distinctive apical structures (arrowheads). Light microscopy with DIC, bar = 0.1 mm. (**e–j**) Detailed views of the apical suction cup-like and finger-like root hair structures. (**e**)(**f**) Light microscopy with DIC, (**g,h,j**) SEM, bars = 25 µm. **(k)** Detail of spirally formed cells walls of root hairs. SEM, bar = 100 µm.

Roots of the majority of the young seedlings (Fig. 1a) were densely covered with root hairs (Fig. 1b) and only a small portion of the examined roots (ca. 10%) did not possess any root hairs. Substrate particles were sometimes attached to the root hairs, often covering the whole root (Fig. 1c). Observations at higher magnifications revealed that the root hairs possessed distinctive apical structures (Fig. 1d). Two forms of these structures were recorded: suction cup-like tips and branched finger-like tips (Fig. 1e–j). Upon detachment of the sampled seedlings, some of the root hairs lost their typical tubular character and the spiral-shaped cell wall became apparent (Fig. 1k).

**Figure 2:**
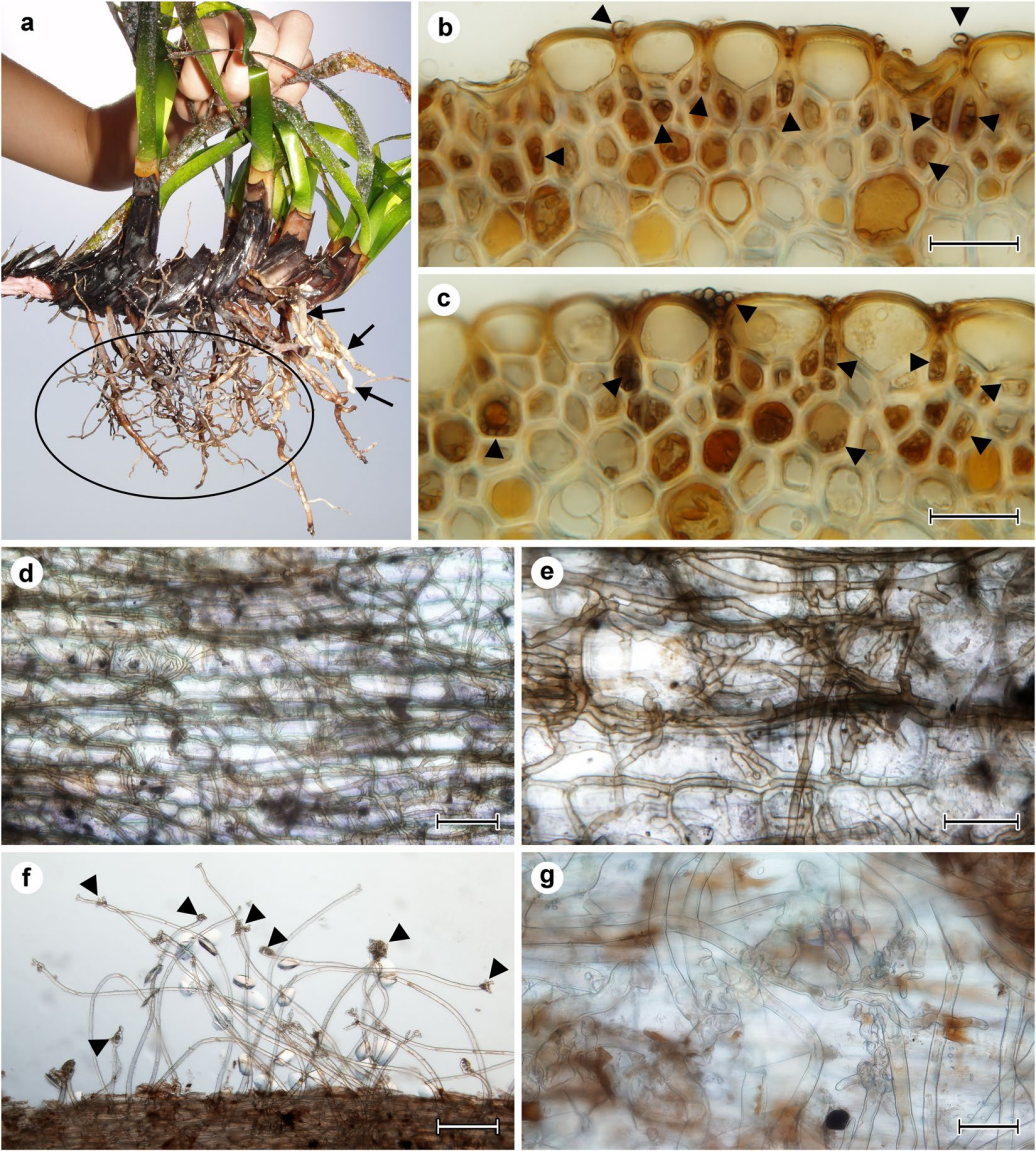
Fungal colonization and root hairs in adult *Posidonia oceanica* plants. (**a**) The lower part of an adult *P. oceanica* plant forming an extensive and branched root system. The typical fungal colonization mostly occurs in the fine terminal roots (ellipse) whereas the thick whitish roots (arrows) are usually free of fungal colonization. (**b**)(**c**) Transversal sections of *P. oceanica* roots colonized by symbiotic fungi (arrowheads). The colonization is confined to the root surface and the hypodermis; both the rhizodermis and the cortex are typically free of any fungal colonization. Light microscopy with DIC, bars = 25 µm. (**d**)(**e**) Dark septate fungi forming loose to dense reticular hyphal nets on the root surface. Light microscopy with DIC, bars 50 µm and 25 µm, respectively. (**f**) Similarly to the seedlings, some adult plants rarely form root hairs with distinctive apical structures (arrowheads). Light microscopy with DIC, bar = 100 µm. (**g**) Detailed view of the apical structures. Light microscopy with DIC, bar = 50 µm.

The young seedlings from the ME-33 site possessed no visible DSE fungal colonization. In contrast, the established seedlings were apparently colonized by dark septate (DS) fungal hyphae occurring mostly on the root surface either in the form of single hyphae or as several parallel hyphae (cf. Fig. 2). The observed fungal colonization anatomically and morphologically corresponded to that described in^28,29^ and^30^ in *P. oceanica* adults in the NW Mediterranean Sea and the central Adriatic Sea, respectively.

When the site with young seedlings (ME-33) was re-visited in September 2017, no seedlings were found at places where they were firstly spotted in September 2012.

### Adult plants

Terminal fine roots of the adult plants (Fig. 2a) typically lacked root hairs but were frequently colonized by DSE fungi. Three forms of root fungal colonization were observed using light microscopy: superficial hyphae, intercellular hyphae in the rhizodermis and the hypodermis and intracellular microsclerotia in the hypodermis (Fig. 2b,c). Occasionally, the hyphae formed dense superficial mycelium which spread over the root as loose to dense hyphal mantles (Fig. 2d,e). Neither the rhizodermal cells nor the cortex and the stele contained DS hyphae. When present (sites ME-65 and 66), root hairs of the adult plants formed both the suction cup-like and the finger-like apical structures (Fig. 2f,g), similarly to the seedlings.

### Statistical analyses

All root traits evaluated in Subset 1 as well as total fungal colonization and superficial mantle-like colonization in Subset 2 showed significant differences between the sampled plant/substrate types (p < 0.05; Tables 1 and 2). In Subset 1, the young seedlings possessed the highest root hair density but were free of visible fungal colonization (Table 1). In contrast, the adults lacked root hairs but displayed the second highest fungal colonization levels which however did not significantly differ from the highest levels recorded in the established seedlings. The established seedlings displayed considerably lower level of root hairs than the young seedlings (Table 1).

**Table 1:** Root hair density and root fungal colonization of *Posidonia oceanica* seedlings and adults in Subset 1.

Sample	Root hair density (number mm^−1^)	Total fungal colonization (%)	Surface hyphae (%)	Intraradical hyphae (%)	Microsclerotia (%)
Generalized linear model	**P** = **4.18 ∗ 10**^**−7**^	**P** = **4.17 ∗ 10**^**−7**^	**P** = **4.85 ∗ 10**^**−7**^	**P** = **9.03 ∗ 10**^**−4**^	**P** = **0.019**
young seedlings (n = 30)	33.4 ± 4.9 **a**	0 **a**	0 **a**	0 **a**	0 **a**
established seedlings (30)	15.7 ± 3.4 **a**	37 ± 5.8 **b**	34 ± 5.4 **b**	9 ± 2.8 **b**	7 ± 2.6 **b**
adult plants (30)	0.00 **b**	33 ± 4.3 **b**	25 ± 3.7 **b**	7 ± 1.3 **b**	12 ± 3.5 **b**

Mean values of root hair density and root fungal colonization (total colonization, colonization by surface hyphae, intraradical hyphae and intracellular microsclerotia) ± SE are given. Significance of the influence of the ontogenetic phase as well as differences between the samples were evaluated and grouped according to the Generalized Linear Model with Gaussian error structure and identity link function. (different letters indicate significantly different groups). For more details see Materials and Methods.

**Table 2:** Root hair density and fungal colonization of *Posidonia oceanica* adults in two different substrates in Subset 2.

Sample	Root hair density (number mm^−1^)	Total colonization (%)	Surface hyphae (%)	Intraradical hyphae (%)	Microsclerotia (%)
Kruskal-Wallis test	H = 1.01	**H** = **8.78**	**H** = **12.29**	H = 1.92	H = 1.13
	p = 0.315	**p** = **0.003**	**p** < **0.001**	p = 0.166	p = 0.289
silty substrate (n = 30)	7.3 ± 3.2	10 ± 3.5 **a**	5 ± 1.9 **a**	5 ± 2.5	3 ± 2.3
sandy substrate (30)	2.8 ± 1.1	20 ± 3.9 **b**	17 ± 3.8 **b**	5 ± 1.6	1 ± 0.8

Mean values of root hair density and fungal root colonization (total colonization, colonization by surface hyphae, intraradical hyphae and intracellular microsclerotia) ± SE are given. Differences between the samples were evaluated using Kruskal-Wallis test and grouped according to the post hoc Nemenyi test (different letters indicate significantly different groups). For more details see Materials and Methods.

In Subset 2, plants from the silty substrate had more than doubled average root hair density than those from the sandy substrate (7.3 ± 3.2 and 2.8 ± 1.1 per 1 mm of the root length, respectively) but this difference was not statistically significant (Table 2). At the same time, plants from the sandy substrate had doubled average total root fungal colonization and more than tripled average colonization by the surface hyphae compared to plants from the silty substrate (Table 2).

The hair root apical structures were recorded in plants from all four sites. More than one third of root segments from the sandy substrate, 60% from the silty substrate, 87% of root segment of the established seedlings and all but one examined root of the young seedlings possessed at least one root hair with the apical structures. A significantly higher number of root hairs possessing the apical structures as well as the proportion of root hairs with the apical structures were recorded in both sets of seedlings compared to the adults (Table 3).

**Table 3:** Frequency of apical root hair structures.

Sample	Density of root hairs with apical structures (number mm^−1^)	Percentage of root hairs with apical structures (out of all root hairs) (%)
Kruskal-Wallis test	**H = 41.61**	**H = 35.10**
	**p < 0.001**	**p < 0.001**
young seedlings (n = 30)	16.6 ± 3.0 **a**	35.8 ± 2.9 **a**
established seedlings (n = 30)	10.8 ± 2.0 **a**	36.3 ± 4.6 **a**
silty substrate (n = 30)	3.3 ± 1.0 **b**	16.3 ± 3.6 **b**
sandy substrate (n = 30)	1.7 ± 0.7 **b**	10.1 ± 3.1 **b**

Mean values of density of root hairs with the distinctive apical structures and fraction of root hairs with the apical structures ± SE are given. Differences between the samples were evaluated using the Kruskal-Wallis test and grouped according to the post hoc Nemenyi test (different letters indicate significantly different groups). For more details see Materials and Methods.

## Discussion

**Figure 3:**
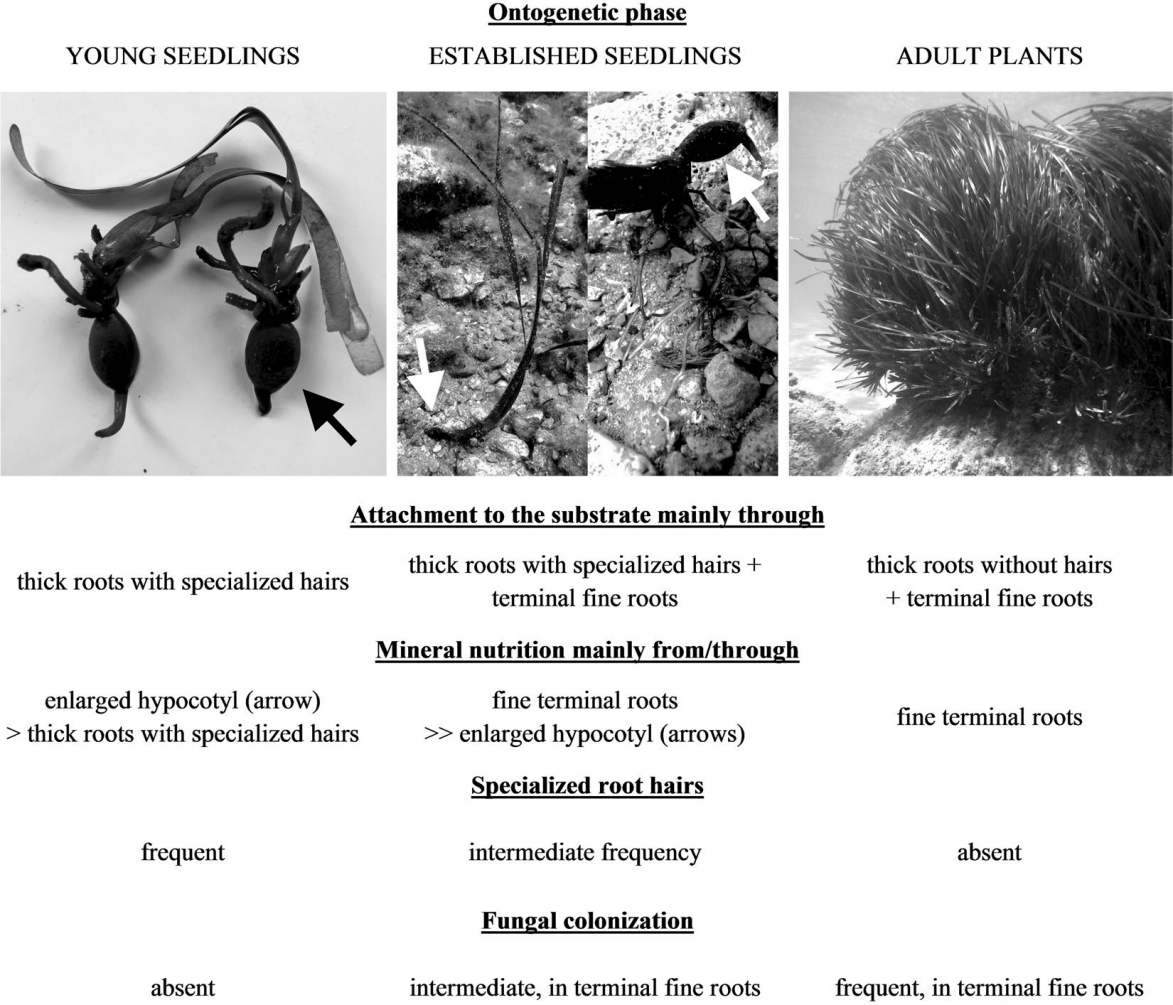
Ontogenetic shift in root traits of the Mediterranean seagrass *Posidonia oceanica*. A graphical summarization of the shift in ecologically significant root traits in *P. oceanica* – a transition from young seedlings possessing dense root hair cover but lacking fungal root colonization to adult plants lacking root hairs but possessing abundant fungal root colonization.

Here we report an intriguing shift in ecologically significant root traits in the dominant Mediterranean seagrass, the endemic *P. oceanica*, as summarized in Fig. 3. To our knowledge, such a transition from young seedlings possessing dense root hair cover but lacking fungal root colonization to adult plants lacking root hairs but possessing abundant fungal root colonization has never been reported before in any seagrass species. While this shift may resemble negative correlation between root hair production and mycorrhizal colonization typical in terrestrial as well as some aquatic plants^8,15,16^, it rather seems to be connected with the seagrass ontogeny and likely reflects different evolutionary pressures/environmental constraints typical for the three studied ontogenetic phases. These mainly comprise the need 1/ to attach to the substrate in the young seedling phase and 2/ to derive mineral nutrients from the substrate in the adult plant phase.

Ad 1/ Adaptations of submerged plants to the permanent movement of water column in the high-energy coastal/shallow lake areas are crucial for their survival and successful growth^51,52^. In seagrasses, these adaptations emerge already during the seed dispersal and seedling establishment ontogenetic phases^22,53^ but seem to be especially important during seedling anchoring^23,54,55^. One such peculiar adaptation in *P. oceanica* concerns the finest parts of its root system, the root hairs. Despite that these have been reported as infrequent/rare^30,39^ to completely absent^24^ in adults, recent studies reveal both regular occurrence and high abundance of root hairs in seedlings as well as their crucial importance for seedling establishment, conferring anchorage strength values up to 5.23 N^23^. These “sticky root hairs” or “adhesive root hairs”^23^ feature specific apical structures as an apparent adaptation to surf exposure^23^, [this study]. Similar apical swellings have already been observed in non-marine environments, e.g., in liverwort rhizoids colonized by the prominent ericoid mycorrhizal fungus *Rhizoscyphus ericae*^56^ which typically inhabits Ericaceae hair roots worldwide^57^, in root hairs of various Podostemaceae which live on naked rocks in waterfalls (“foot-like broadened tips”^58^) and in root hairs of aerial roots of *Syngonium podophyllum* (Araceae) (“hair roots with swollen tips”^59^). Greater abundance of the adhesive apical structures as well as higher proportion of root hairs with these structures recorded in seedlings compared to adult plants support the assumption that adhesion to the substrate provided by root hairs is significant for survival of *P. oceanica* seedlings but continuously loses its importance in later ontogenetic phases.

**Figure 4:**
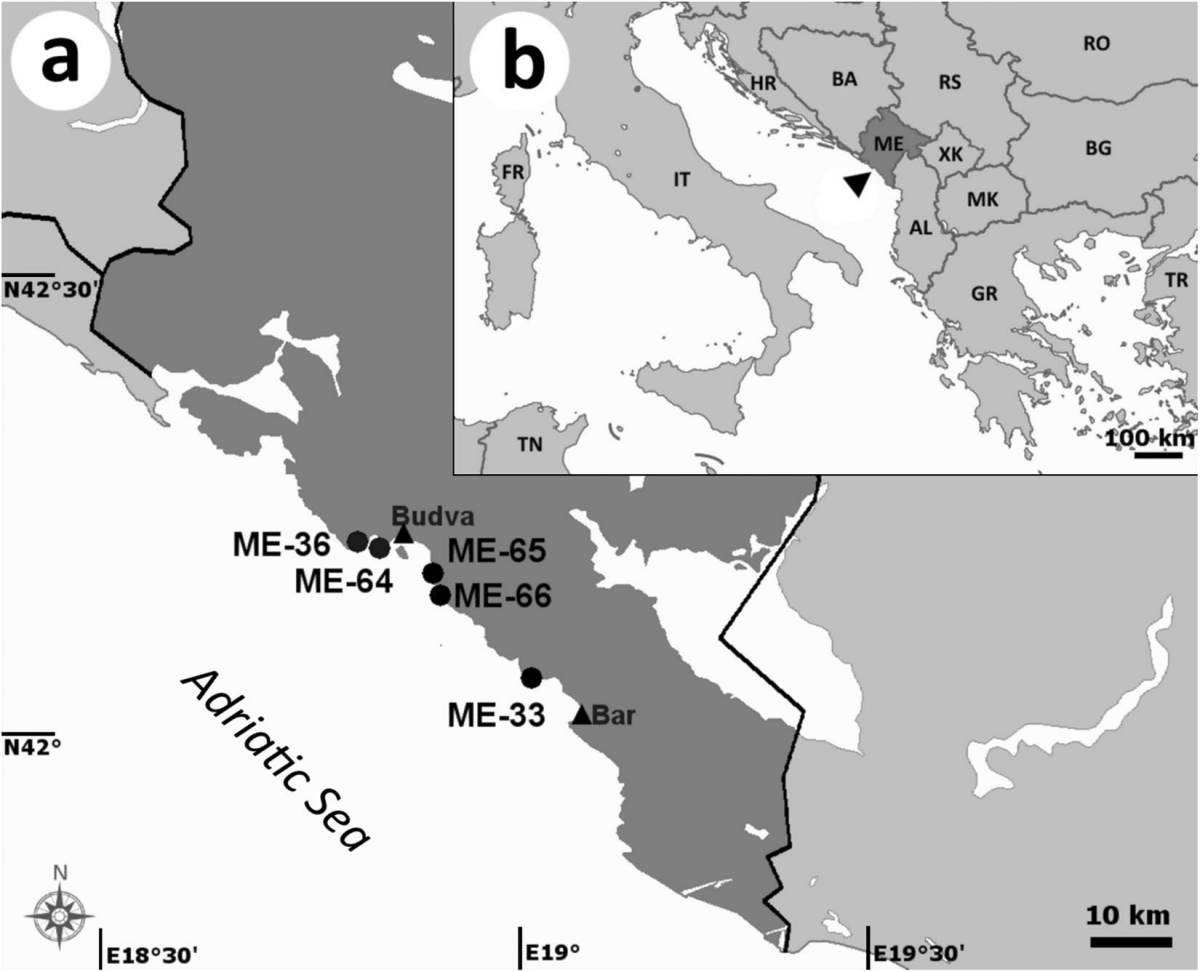
Location of the *Posidonia oceanica* sites sampled in this study. (**a**) The location of the sampling sites (dots) along the central coast of Montenegro, in the vicinity of the cities of Bar and Budva (triangles). For site names and their GPS coordinates see Table 1. Bar = 10 km. (**b**) The location of Montenegro (arrowhead) within the Adriatic region and its surroundings. Two-digit country codes follow the ISO 3166–1 alpha-2 standard, XK stands for Kosovo; bar = 100 km.

In addition to the apical swellings, *P. oceanica* root hairs are strengthened by spirally formed cells walls^30^, [this study], a feature recently reported also by Tomasello *et al*.^60^. These authors recognized two types of root hairs in *P. oceanica* growing in NW Sicily (Italy), i.e., “tubular” and “spiral-shaped”, where the latter is morphologically identical to the elongated root hairs probably damaged by too high breaking force as reported here and in Vohník *et al*.^30^ (compare Fig. 1h,i, page 46 in Tomasello *et al*.^60^ with Fig. 2o, page 113 in Vohník *et al*.^30^ and Fig. 1k in this study). Similar feature has been recently reported also in some orchids by Bernal *et al*.^61^ (“spiral root hairs”; see Figs 2–6, page 414) and Almeida *et al*.^62^ (“spiral-shaped root hairs”; see Fig. 1g,h, page 124). However, we suggest that instead of representing morphologically distinct root hairs, the “spiral” root hairs represent cracked tubular root hairs, in contrast to, e.g., the shoehorn or spiral root hairs with spoon-like tips reported in *Hedera helix* (Araliaceae) by Melzer *et al*.^63^. Indeed, the spirally formed cell walls in *P. oceanica* root hairs are morphologically identical with the helical cell walls in *S*. *podophyllum* (“helical ribbon-like root hairs”; see Fig. 5, page 513, Fig. 8, page 515 and Fig. 10, page 516 in Yang & Deng^59^) which “serve as energy-dissipating units to ensure the safe linkage between the root and substrate” (Yang & Deng^59^, page 508).

To our knowledge, the significance of root hairs in *P. oceanica* nutrient uptake has not been rigorously evaluated but seems to be secondary to their role in seedling attachment to the substrate. Indeed, *P. oceanica* seedlings may rely on the nutrients stored in the enlarged hypocotyl for up to several months after their germination^64^. With progressing time, along with depletion of nutrients from the seed, the roots take over the role in providing majority of nutrition to the plantlets^40^ and maintain their dominant position in the nutrition of the adult plants^20^. Our observation that the production of root hairs decreased in older, established seedlings and especially in adults suggests that with progressing time, the newly formed fine roots take over also the anchoring function.

Intriguingly, while the production of root hairs is reduced in established seedlings and especially adults, their terminal fine roots become colonized by DS mycelium thus forming the specific root-fungus symbiosis recently recorded in *P. oceanica* adults at many localities in the NW Mediterranean and the Central Adriatic^28–30^. Eventually, a major part of the *P. oceanica* root system may be formed by these often heavily colonized roots. In contrast, this symbiosis was absent in the young seedlings and there are at least three possible explanations: i/ the respective mycobiont(−s) were missing at the exposed microsites colonized by the young seedlings, ii/ young *P. oceanica* seedlings possess some mechanisms which prevent them from establishing root fungal symbioses as long as they can utilize their own reserves, and iii/ establishment of the specific root-fungus symbiosis is a lengthy process hence this association cannot be detected in early phases of *P. oceanica* ontogeny. However, our data are too few to allow any definite conclusions with respect to these possibilities.

Ad 2/ Considering that the often heavily colonized terminal fine roots likely represent a site of significant nutrient uptake by *P. oceanica*, it is tempting to speculate about the role of the specific fungal symbiosis in facilitation of the nutrient influx from the seabed substrate/matte to the seagrass.

Firstly, seagrasses represent a secondary adaptation to the marine environment, i.e., their ancestors were terrestrial plants. Secondly, since the great majority of terrestrial plants rely on mycorrhizae in nutrient uptake from recalcitrant substrates, it is reasonable to suppose that the ancestors of seagrasses were mycorrhizal. Thirdly, the fungal colonization on the root surface in adult *P. oceanica* plants resembles, at least to some extent, sparse hyphal mantles formed by some contact exploration ectomycorrhizal fungi^30^. On the other hand, a specific plant-fungus interface for nutrient exchange, analogous to arbuscules, the Hartig net or intracellular hyphal coils and pelotons common in terrestrial mycorrhizae, is missing. Fourthly, mycorrhizal colonization is typically negatively correlated with root hair production. On the other hand, also colonization by endophytes with DS hyphae may be negatively correlated with root hair production^65^ and these endophytes typically do not take part in nutrient uptake by the host. What is then the eco-physiological functioning of the specific fungal symbiosis in *P. oceanica* roots? Because the complete lack of regular *P. oceanica* seed supply and the very complicated growth of the dominant pleosporalean mycobiont in pure culture^29^ make manipulative inoculation experiments practically impossible, we can only hypothesize. Perhaps the DS fungi colonizing *P. oceanica* roots cheat the ancient mechanisms evolved during the long co-evolution of land plants and their root mycobionts^66^ by utilizing photosynthetically bound carbon but providing no reciprocal reward to the seagrass (i.e., they behave as biotrophic parasites). Alternatively, this presumably endophytic fungal association is a prelude to evolution of some kind of marine mycorrhizal symbiosis; according to the so-called waiting room hypothesis, some mycorrhizal fungal lineages evolved from former root endophytes, because root endophytism acts as a symbiotic “waiting room” enabling the interacting partners to evolve a tighter mutualistic relationship^67^. To conclude, while there is no solid proof for the involvement of the pleosporalean fungus in the seagrass nutrient uptake, the regular occurrence of the specific root-fungus symbiosis formed by a mycobiont not known from other hosts or environments in the dominant Mediterranean seagrass is intriguing and begs further investigation.

When revisiting the Sutomore sampling site (ME-33), no seedlings were found in September 2017 at the original microsites. Since the seedlings found here in September 2012 occupied rather shallow depths, this observation is in agreement with the results of Piazzi *et al*.^49^ where all seedlings on rocks and gravel settled in the depth of 2 m eventually died. In contrast, Alagna *et al*.^55^ reported successful seedling establishment and survival in 1–3 m depths which probably reflects different hydrodynamic conditions at the studied localities. Hypothetically, the observed die off could be at least in part caused by a lack of compatible DS mycobionts at the exposed microsites colonized by the young seedlings or by the very slow growth of the main DS mycobiont, the Pleosporales sp. MV-2012^29,30^.

## Methods

### Sampling

Root samples of young seedlings, older and larger already established seedlings and adult plants of *Posidonia oceanica* (L.) Delile were collected at 5 sites in the southern Adriatic Sea off Montenegro by scuba diving in September 2012 and August 2014 (Table 4, Fig. 4). Additionally, survival of the young seedlings was checked in September 2017. Thirty young seedlings firmly attached to submerged rocks in shallow water (0.5–2 m) under a steep cliff were randomly sampled at the first site (ME-33) while 30 established seedlings growing in deeper water (10–12 m) were randomly sampled at the second site (ME-64). Control adult plants were randomly collected at the third site (ME-36) at the depth of approximately 6 m. For screening of the effect of the substrate type on the root hair density and root fungal colonization, adult plants were randomly collected at a site with silty seabed substrate (ME-65) and at a site with sandy seabed substrate (ME-66). Whole seedlings were carefully detached from the seabed and inserted into 1500 ml plastic flasks with seawater while root bulk samples of adult plants (5 plants at least 5 m apart) were inserted into 50 ml plastic beakers with seawater. Subsequently, seawater was substituted with 30% ethanol and both the flasks and the beakers were transported to the laboratory where they were stored in a fridge (5–8 °C) until processed.

**Table 4:** *Posidonia oceanica* sites sampled in this study.

Site #^a^	Sampling subset	Sampling site name	GPS coordinates	Sampling date	Sampling depth	*Posidonia oceanica* phenotype/substrate
ME-33	1	Sutomore, Crni Rtič (Black Cape)	N42.13595, E19.01549	September 2012^b^	0.5–2 m	young seedlings (n = 30)
ME-36	1	Krimovica, Trsteno Beach I	N42.27985, E18.78738	August 2014	~6 m	adult plants (bulk samples from 5 specimens)
ME-64	1	Krimovica, Trsteno Beach II	N42.27336, E18.80058	August 2014	10–12 m	established older seedlings (30)
ME-65	2	Sveti Stefan II	N42.25311, E18.89444	August 2014	~2.5 m	adult plants, silty substrate (bulk samples from 5 specimens)
ME-66	2	Sveti Stefan III	N42.25244, E18.89578	August 2014	~3.5 m	adult plants, sandy substrate (bulk samples from 5 specimens)

^a^The numbering of the sites continues from Vohník *et al*.^28–30^.

^b^This site was re-visited in September 2017 and no seedlings were found.

### Microscopic observations

Thirty approximately 1 cm long root segments were randomly selected from all samples from all sites and screened using an Olympus SZX12 stereomicroscope. Root hair density was calculated as the number of visible root hairs per 1 mm of the root segment length. Semi-thin transversal sections of the root segments prepared using a manual microtome were screened at high magnifications (200–400x) using an Olympus BX60 microscope equipped with Differential Interference Contrast (DIC). Root fungal colonization was estimated separately for superficial hyphae and two types of intraradical colonization structures – microslerotia and intercellular hyphae. Superficial hyphal colonization was calculated as a percentage of the rhizodermal cells with presence of fungi on their surface. Both types of intraradical colonization were estimated as a percentage of the hypodermal cells containing the respective fungal structure. Additionally, total sum of fungal colonization was calculated. Each root segment was divided into sections so that rhizodermal cells and hypodermal cells directly beneath it formed one section. If fungal structures were present either on the surface or in the hypodermis, the section was scored for fungal presence. Fraction of sections with fungal presence out of all sections summed up total fungal colonization.

Some root hairs possessed distinctive apical structures. From each sampling site with recorded root hair presence, thirty root segments possessing root hairs were randomly selected and used for an estimation of the frequency of the apical structures. The number of root hairs possessing apical structures per 1 mm of the root length was counted and the fraction of the root hairs possessing the apical structures was calculated.

Photographic documentation was obtained using an Olympus DP70 camera and the image processing software Quick PHOTO MICRO 2.3 (Promicra Ltd., Czech Republic). Scanning electron microscopy (SEM) was performed using a FEI Quanta 200 scanning electron microscope in the ESEM mode at low temperatures (−12 °C to −3 °C). Photographs were subsequently modified for clarity in Paint.NET (https://www.getpaint.net/index.html) as needed and assembled using the same software. For more details on microscopic techniques and photo processing see Vohník *et al*.^68^.

### Statistical analyses

For the purpose of statistical analyses, root samples were divided into two Subsets. Subset 1 consisted of young seedlings (sampling site ME-33) and established seedlings (ME-64) together with adult plants from the site ME-36. Subset 2 comprised adult plants from both the silty (ME-65) and sandy (ME-66) substrates at Sveti Stefan (Table 1). For fungal colonization and root hair density values, homogeneity of variances was tested using the Leven’s test; normality of the distribution was tested using the Shapiro-Wilk test. Subsequently, in order to determine the influence of the type of the seabed substrate on the root fungal colonization and the density of the root hair cover in Subset 2, the non-parametric Kruskal-Wallis test was carried out. In the case of significant differences between the groups (p < 0.05), the multiple comparison post-hoc Nemenyi test was performed. In Subset 1, no root hair presence was recorded in adult plants and no fungal colonization was observed in the roots of young seedlings. In order to avoid zero inflation of results, we determined the influence of the plant ontogenetic phase on the root fungal colonization and the density of the root hair cover in Subset 1 using Generalized linear model with Gaussian error structure and identity link function.

Frequency of the apical root hair structures and fraction of the root hairs possessing the apical structures were evaluated in a separate analysis using methods identical to those used for Subset 2. In this analysis, all four groups with recorded root hair presence (i.e., young seedlings, established seedlings and adult plants from both the silty and the sandy substrate) were analyzed as a single dataset.

All statistical analyses were carried out using the R software ver. 3.3.2. For calculation of the Nemenyi test, the PMCMR package was employed^69^.

### Data availability

The datasets generated during the current study are available from the corresponding author on reasonable request.
